# Carglumic Acid Contributes to a Favorable Clinical Course in a Case of Severe Propionic Acidemia

**DOI:** 10.1155/2020/4709548

**Published:** 2020-03-09

**Authors:** Jun Kido, Shirou Matsumoto, Kimitoshi Nakamura

**Affiliations:** Department of Pediatrics, Graduate School of Medical Sciences, Kumamoto University, Kumamoto, Japan

## Abstract

Propionic acidemia (PA) is manifested as an abnormal accumulation of propionic acid and its metabolites, including methylcitrate, 3-hydroxypropionic acid, and propionylglycine, and is caused by a defect of propionyl-CoA carboxylase. PA is complicated by acute life-threatening metabolic crises, which are precipitated by a catabolic state and result in multiple organ failure or even death, if untreated. A neonate with PA recovered from the first metabolic crisis 3 days after birth but developed a second metabolic crisis during the recovery phase. This patient was considered to have severe PA and was accordingly given carglumic acid treatment in combination with carnitine supplementation and protein restriction, which was expected to prevent a recurrent metabolic attack. The patient did not develop hyperammonemia after receiving carglumic acid and was never hospitalized. Moreover, she did not present with acidosis even during viral infection. At 26 months of age, she led a stable life while receiving carglumic acid and regular rehabilitation. Carglumic acid treatment in combination with carnitine supplementation and protein restriction prevented metabolic decompensation, which would have otherwise required hospitalization, and resulted in improved quality of life and developmental outcomes.

## 1. Introduction

Propionic acidemia (PA) (MIM number: 606054) is an autosomal recessive genetic disease that affects the catabolic pathways of the branched-chain amino acids valine and isoleucine, methionine, threonine, thymine, odd-chain fatty acids, and the side chain of cholesterol. It is characterized by an abnormal accumulation of propionic acid and its metabolites, including 3-hydroxypropionic acid, propionylglycine, and methylcitrate, and is caused by a defect of propionyl-CoA carboxylase (PCC) (EC: 6.4.1.3) that converts propionyl-CoA to methylmalonyl-CoA [1].

Acute life-threatening metabolic crises complicate PA. These are precipitated by a catabolic state and result in multiple organ failure or even death, if untreated [1]. The age of onset and clinical course vary among patients, with symptoms including neonatal metabolic encephalopathy, recurrent ketoacidotic coma or Reye-like syndromes, psychomotor retardation, and failure to thrive, in the absence of acute crises.

Prevention of catabolism in the body via the administration of intravenous fluids containing glucose is critical in managing PA metabolic crises. In hyperammonemia, protein intake is restricted, and medications used in urea cycle disorders including l-arginine-HCl, sodium phenylbutyrate, sodium benzoate, and carglumic acid are administered. Persistent hyperammonemia, metabolic acidosis, and severe electrolyte imbalances are indications for extracorporeal detoxification such as plasma exchange and continuous hemodiafiltration (CHDF) [2].

Here, we describe the case of a neonate with PA who recovered from the first metabolic crisis 3 days after birth and developed a second crisis during the recovery phase. We describe the clinical course of the patient following the administration of carglumic acid and discuss its role in her treatment.

## 2. Case Presentation

A 6-day-old female neonate was admitted to our institution with metabolic acidosis and hyperammonemia. She was born at 40 weeks and 2 days' gestation and weighed 2828 g. Her APGAR scores at 1 and 5 minutes after birth were 9 and 10, respectively. There were no fetal or maternal medical problems during pregnancy. From day 3 after birth, her feeding gradually decreased, she could not feed on breast milk, and she developed metabolic acidosis (pH: 7.26, HCO_3_^−^: 13.3 mmol/L, BE: −12.3 mmol/L, anion gap (AG): 19.7) and hyperammonemia (881 *μ*mol/L). She accordingly underwent CHDF and treatment for hyperammonemia, including the administration of arginine, sodium benzoate, L-carnitine, biotin, and multiple vitamins such as vitamins B1, B6, B12, C, and coenzyme Q10 in the intensive care unit. Her blood ammonia level decreased to 435 *μ*mol/L six hours after undergoing CHDF but increased to 739 *μ*mol/L 12 hours after undergoing CHDF. Additional treatment was, therefore, needed, and citrulline and sodium butyrate were administered. Her blood ammonia levels decreased with time, and CHDF was discontinued after 74 hours. The blood ammonia levels did not exceed 60 *μ*mol/L thereafter, and she recovered from the metabolic crisis. We diagnosed her with PA owing to the large quantities of 3-hydroxypropionic acid, methyl citric acid, propionylglycine, and 2-methyl-3-hydroxybutyric acid excreted in the urine. Ten days after withdrawal of CHDF, she developed a second metabolic crisis with severe acidosis and apnea (pH: 6.73, pCO2: 89.9 mmHg, HCO_3_^−^: 11.5 mmol/L, BE: −24.6 mmol/L, AG: 33.1, lactate: 9.00 mmol/L, pyruvate: 0.46 mmol/L, lactate/pyruvate ratio: 19.6) without hyperammonemia (54 *μ*mol/L). We administered carglumic acid (100 mg/kg/day) to prevent secondary hyperammonemia. She recovered from the second metabolic crisis after 3 days without CHDF on infusion of glucose, L-carnitine, carglumic acid, multivitamins, potassium citrate, and sodium citrate. We continued administering these medicines and special formulas for protein restriction after she recovered from the crisis.

**Figure 1:**
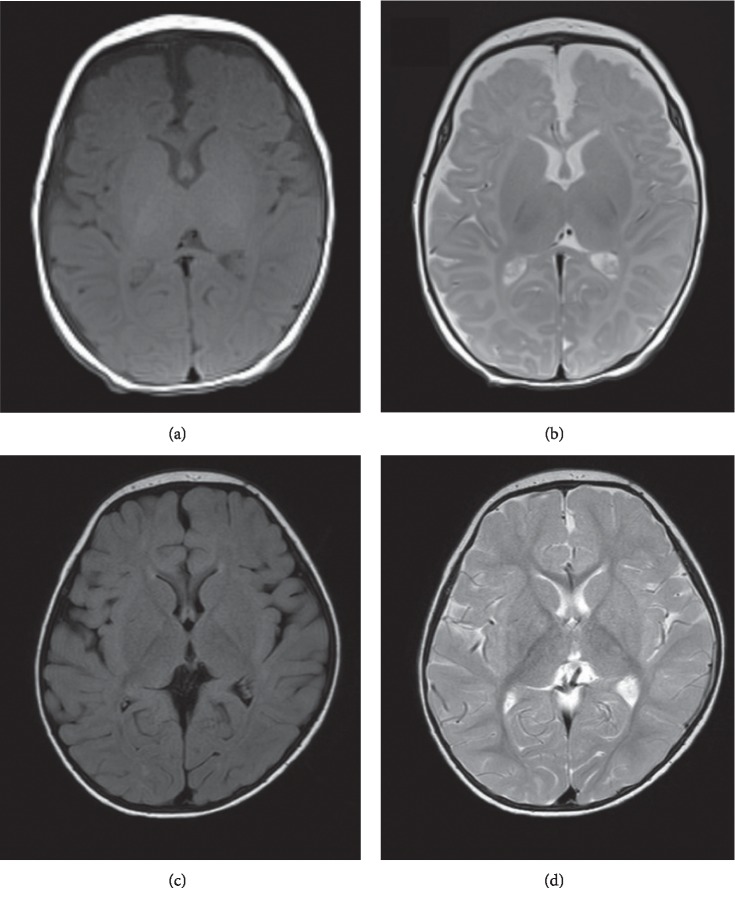
Brain magnetic resonance imaging in the patient with severe propionic acidemia. (a) T1-weighted imaging at the age of 2 months, (b) T2-weighted imaging at the age of 2 months, (c) T1-weighted imaging at the age of 23 months, and (d) T2-weighted imaging at the age of 23 months.

She did not develop hyperammonemia after discharge and was never hospitalized under carglumic acid treatment. Moreover, she did not present with acidosis even during a viral infection (Table 1). Her genetic analysis revealed a *c.923dupT (p.Leu308fs)* homozygous mutation in the *PCCA gene.* Analysis of her blood acylcarnitine and amino acids using tandem mass spectrometry while receiving combination therapy with carglumic acid, L-carnitine, and a restricted protein diet demonstrated a lowered blood propionylcarnitine (C3) level, C3/acetylcarnitine (C2) ratio, and valine and isoleucine + leucine levels (Tables 2 and 3). Brain magnetic resonance imaging at the age of 2 months revealed mild atrophy in the frontal lobe; however, imaging at the age of 23 months did not reveal brain atrophy (Figure 1). At the age of 24 months, her development quotient (DQ) on the Kyoto Scale of Psychological Development corresponded to that at the age of 12 months in a healthy control (total DQ: 49). At 26 months of age, she was leading a stable life while receiving carglumic acid (50 mg/kg/day) and regular rehabilitation.

## 3. Discussion

We presented a case of severe PA in a neonate, who developed a metabolic crisis with severe hyperammonemia and acidosis. The *c.923dupT (p.Leu308fs)* homozygous mutation in the *PCCA* gene resulted in null propionyl-CoA carboxylase activity and led to the development of a severe type of PA [3, 4].

Hyperammonemia ≥360 *μ*mol/L has a significant adverse effect on the brain and is likely to result in mental retardation [5]. Therefore, in PA with metabolic decompensation, any excess toxic metabolites and ammonia should be removed from the body as soon as possible. Moreover, it is important to manage patients with PA to prevent metabolic acidosis and hyperammonemia in the long term, as far as practicable [2].

In patients with PA, excessive propionyl-CoA inhibits N-acetylglutamate synthase (NAGS) [6], which catalyzes the formation of NAG, an activator of carbamoyl phosphate synthetase I (CPSI). CPSI is a key enzyme in the first step of the urea cycle. Propionyl-CoA also inhibits the pathway by depleting hepatic acetyl CoA, which is responsible for NAG synthesis. Carglumic acid is a synthetic structural analogue of NAG and is, therefore, specifically indicated for treating hyperammonemia in patients with PA. In 2016, the Ministry of Health, Labour and Welfare approved its clinical use in Japan for the treatment of hyperammonemia owing to primary NAGS deficiency and organic acidemia. Carglumic acid accelerates ammonia detoxification by mimicking the effects of NAG on CPSI, thereby driving the urea cycle forward independent of other mechanisms that detoxify organic acids.

There are some reports on the long-term treatment of NAGS deficiency, and the short- and acute-term treatment of organic acidemia for hyperammonemia [7, 8]. However, the long-term effect of treatment in patients with organic acidemia remains unknown. The clinical course, blood C3 level, and C2/C3 ratio in this case suggested the effectiveness of long-term carglumic acid treatment in patients with severe PA. Blood acylcarnitine or amino acid levels exceeding the cutoff values indicate metabolic disorders of fatty, amino, and organic acids [9, 10], and the blood C3 level and C3/C2 ratio significantly increase in severe PA.

The urea cycle is linked to the tricarboxylic acid (TCA) cycle. Fumarate, synthesized form argininosuccinate in the urea cycle, is utilized in the TCA cycle. Impaired ammonia detoxification in the urea cycle leads to impaired function of enzymes in the TCA cycle owing to ammonia toxicity [11] and a shortage of substrates in the TCA cycle.

Regulation of the urea cycle, therefore, prevents dysfunction in the TCA cycle. Moreover, carglumic acid may be converted to *α*-ketoglutaric acid, which is utilized in the TCA cycle via anaplerosis. Although carglumic acid is considered to increase substrates in the TCA cycle via anaplerosis, succinyl-CoA may not be necessarily decreased. Furthermore, the reverse conversion of succinyl-CoA to propionyl-CoA is minimal. The beneficial impact of carglumic acid on the TCA cycle could result from compensatory anaplerosis following the lack of anaplerotic contribution from propionyl-CoA in PA. Carglumic acid, therefore, contributes to the regulation of urea and TCA cycles and prevents the excessive production and accumulation of propionic acid and its metabolites. In this report, we described the outcome of treatment of severe PA with carglumic acid for 2 years. It was considered that her good clinical course and blood C3 level and C3/C2 ratio could be ascribed to carglumic acid in combination with protein restriction and L-carnitine. Protein restriction, L-carnitine supplementation, or carglumic acid treatment alone may not achieve good clinical outcomes in severe cases of PA. Cumulative evidence from further cases is needed to evaluate the effects of carglumic acid treatment for longer duration in PA.

In conclusion, carglumic acid combined with the standard treatments such as protein restriction and L-carnitine prevents metabolic decompensation, which would otherwise require hospitalization, and results in improved quality of life and developmental outcomes. The effects of carglumic acid should be given serious consideration, and possible indications for its long-term administration in severe organic acidemias should be explored.

## Figures and Tables

**Table 1:** Venous blood gas analysis at the time of discharge and at 1, 3, 6, and 12 months after discharge.

	At the time of discharge	1 month	3 months	6 months	12 months
PH	7.35	7.39	7.40	7.36	7.43
BE (mmol/L)	−2.1	−2.7	−5.1	−0.9	−3.3
HCO_3_^−^ (mmol/L)	23.4	22.0	19.0	23.8	20.2
NH_3_ (*μ*mol/L)	25	41	33	36	59

**Table 2:** Blood acylcarnitine analysis before and after carglumic acid and carnitine treatment.

	At the onset time (before carglumic treatment)	3 months after carglumic treatment	Cutoff values (*μ*mol/L)
C0	23.96	42.78	<10.00
C2	10.76	24.91	
C3	73.85	9.78	≥3.50
C3/C2	6.86	0.39	≥0.25
C4	0.32	0.29	≥0.60
C5	0.21	0.2	≥1.00
C5 : 1	0.04	0.01	≥0.025
C5DC	0.08	0.06	≥0.25
OH-C5	0.19	0.24	≥0.60
C6	0.08	0.08	
C8	0.09	0.06	≥0.30
C8/C10	1	0.79	≥1.00
C10	0.09	0.07	≥0.40
C12	0.09	0.11	
C14	0.1	0.28	
C14 : 1	0.07	0.05	≥0.40
C16	0.97	1.73	≥3.00
OH-C16	0.01	0.02	≥0.05
C0/(C16+C18)	18.75	17.25	≥100.00
C18	0.31	0.76	
C18 : 1	0.47	1.02	
OH-C18 : 1	0.01	0.01	≥.0.05

C0: free carnitine; C2: acetylcarnitine; C3: propionylcarnitine; C4: butyrylcarnitine; C5: isovalerylcarnitine; C5:1: tiglylcarnitine; C5-DC: glutarylcarnitine; OH-C5: 3-hydroxy isovalerylcarnitine; C6: hexanoylcarnitine; C8: octanoylcarnitine; C10: decanoylcarnitine; C12: lauroylcarnitine; C14: myristoylcarnitine; C14 : 1: myristoylcarnitine; C16: palmitoylcarnitine; OH-C16: 3-hydroxy palmitoylcarnitine; C18: octadecanoylcarnitine C18 : 1: octadecenoylcarnitine; OH-C18 : 1: 3-hydroxy octadecenoylcarnitine.

**Table 3:** Blood amino acids analysis before and after carglumic acid and carnitine treatment.

	At onset (before carglumic treatment)	3 Months after carglumic treatment	Cutoff values (mg/dL)
Valine	1.62	0.25	≥2.93
Methionine	0.21	0.07	≥1.20
Isoleucine + leucine	1.14	0.58	≥4.59
Tyrosine	0.77	0.26	≥8.00
Phenylalanine	0.48	0.33	≥3.00
Citrulline	0.13	0.18	≥0.88
Arginine	0.24	0.2	≥2.61

## References

[1] Fenton W. A., Gravel R. A., Rosenblatt D. S., Scriver C. R., Beaudet A. L., Sly W. S. (2001). Disorders of propionate and methylmalonate metabolism.. *The Metabolic and Molecular Bases of Inherited Disease*.

[2] Baumgartner M. R., Hörster F., Dionisi-Vici C. (2014). Proposed guidelines for the diagnosis and management of methylmalonic and propionic acidemia. *Orphanet Journal of Rare Diseases*.

[3] Pérez B., Desviat L. R., Rodríguez-Pombo P. (2003). Propionic acidemia: identification of twenty-four novel mutations in Europe and North America. *Molecular Genetics and Metabolism*.

[4] Gallego-Villar L., Pérez-Cerdá C., Pérez B. (2013). Functional characterization of novel genotypes and cellular oxidative stress studies in propionic acidemia. *Journal of Inherited Metabolic Disease*.

[5] Kido J., Nakamura K., Mitsubuchi H. (2012). Long-term outcome and intervention of urea cycle disorders in Japan. *Journal of Inherited Metabolic Disease*.

[6] Coude F. X., Sweetman L., Nyhan W. L. (1979). Inhibition by propionyl-coenzyme A of N-acetylglutamate synthetase in rat liver mitochondria. A possible explanation for hyperammonemia in propionic and methylmalonic acidemia. *Journal of Clinical Investigation*.

[7] Valayannopoulos V., Baruteau J., Delgado M. B. (2016). Carglumic acid enhances rapid ammonia detoxification in classical organic acidurias with a favourable risk-benefit profile: a retrospective observational study. *Orphanet Journal of Rare Diseases*.

[8] Häberle J. (2011). Role of carglumic acid in the treatment of acute hyperammonemia due to N-acetylglutamate synthase deficiency. *Therapeutics and Clinical Risk Management*.

[9] Tajima G., Sakura N., Yofune H. (2005). Enzymatic diagnosis of medium-chain acyl-CoA dehydrogenase deficiency by detecting 2-octenoyl-CoA production using high-performance liquid chromatography: a practical confirmatory test for tandem mass spectrometry newborn screening in Japan. *Journal of Chromatography B*.

[10] Rinaldo P., Cowan T. M., Matern D. (2008). Acylcarnitine profile analysis. *Genetics in Medicine*.

[11] Katunuma N., Okada M., Nishii Y. (1966). Regulation of the urea cycle and TCA cycle by ammonia. *Advances in Enzyme Regulation*.

